# Microbial response mechanisms of organic nitrogen substitution for optimizing tobacco yield and quality: the key role of 50% organic nitrogen

**DOI:** 10.3389/fmicb.2025.1698745

**Published:** 2025-10-09

**Authors:** Yongjin Liang, Wuyang Cheng, Bo Peng, Jinglin Xiao, Yi He, Heyou Xiao, Rongcheng Dai, Qiu Huang, Fei Chen, Huarong Ling, Shijie He, Ruixuan Zhu, Jianyu Wei

**Affiliations:** ^1^China Guangxi Tobacco Industry Co., Ltd., Nanning, Guangxi, China; ^2^China Hunan Tobacco Industry Co., Ltd., Changsha, Hunan, China; ^3^Shaoyang Tobacco Company, China Tobacco Hunan Industrial Co., Ltd., Shaoyang, Hunan, China; ^4^Nanxiong Tobacco Company, China Tobacco Guangdong Industrial Co., Ltd., Nanxiong, Guangdong, China; ^5^Liuzhou Cigarette Factory, China Tobacco Guangxi Industrial Co., Ltd., Liuzhou, Guangxi, China; ^6^Hezhou Tobacco Factory, China Tobacco Guangxi Industrial Co., Ltd., Hezhou, Guangxi, China; ^7^College of Agriculture, Guangxi University, Nanning, Guangxi, China

**Keywords:** tobacco, organic nitrogen substitution, soil microbial communities, 50% substitution ratio, yield and quality

## Abstract

**Introduction:**

Precise matching of nutrient supply with plant demand in tobacco cultivation is crucial for achieving high yield and superior quality. Traditional chemical fertilizer application modes have obvious limitations, and although organic nitrogen substitution has become an important development direction, the determination of optimal substitution ratios and their microbial regulatory mechanisms still require in-depth research.

**Methods:**

Based on previous studies, this research established refined organic nitrogen substitution experiments with treatments of 40, 50, and 60% organic nitrogen substitution at two experimental sites, systematically evaluating the effects of different substitution ratios on yield and quality of tobacco, while analyzing differences in soil microbial community composition, function, and network correlations through 16S rRNA sequencing and network analysis.

**Results:**

Results showed that organic nitrogen treatments significantly improved the agronomic traits, yield performance, chemical composition, and sensory quality of tobacco. The underlying microbial community mechanisms revealed that organic nitrogen application significantly enhanced soil microbial community diversity, enriched beneficial bacterial groups (Pseudomonadota, Actinomycetota, Bacteroidota, etc.), strengthened carbon and nitrogen cycling functions, and increased network complexity. Redundancy analysis demonstrated that the microbial communities under organic nitrogen treatments were closely associated with yield and quality traits such as potassium and total sugars. Among all treatments, the 50% organic nitrogen treatment performed optimally, achieving yield increases of 63.4 and 67.8% at two experimental sites respectively, with the best tobacco leaf chemical quality and sensory characteristics. This treatment also exhibited superior performance in microbial community structure and functional coordination, and the study found that 50% is the optimal substitution ratio for microbial response.

**Discussion:**

This study confirmed that 50% organic nitrogen substitution constitutes the optimal fertilization scheme and revealed the underlying microbial response mechanisms by which this ratio optimizes tobacco quality, providing scientific guidance for precision fertilization of tobacco based on microbial theory.

## 1 Introduction

The quality formation of tobacco (*Nicotiana tabacum* L.) highly depends on the precise balance of various chemical components within the leaves (Ullah et al., 2025). Sugars provide sweet aromatic flavors during smoking (Talhout et al., 2006), nicotine content determines the irritation and satisfaction experienced during smoking (Sansone et al., 2023), and potassium influences combustion characteristics (Hu et al., 2021). The content and ratios of these key components are directly regulated by the rhizosphere soil microenvironment around plant roots. Tobacco leaves can sensitively respond to subtle changes in soil nutrient supply and translate these into significant differences in quality (Teng et al., 2024). In current tobacco production, optimizing the proportion of leaf chemical components while ensuring yield has become the core objective for enhancing tobacco commercial value. The quality control mechanism of tobacco manifests as direct transmission from soil characteristics to leaf quality. Therefore, cultivation management places extremely high demands on soil nutrient regulation, requiring not only satisfaction of basic nutrient requirements for plant growth and development, but also precise matching between the timing and intensity of nutrient supply and plant metabolic rhythms. Any deviation in these processes may lead to leaf quality decline or imbalance (Zheng et al., 2021).

Traditional chemical fertilizer application modes simplify the complex soil–plant system into a simple “input–output” relationship. Although this simplification can rapidly replenish soil nutrients in the short term, it overlooks that soil is not simply a nutrient reservoir, but rather an active ecosystem involving microorganisms (Bardgett and van der Putten, 2014). The rapid and massive release of chemical fertilizers seriously disrupts the normal functions of soil microbial communities and destroys the long-established coordination mechanisms between plants and soil microenvironments (Geisseler and Scow, 2014). Under this forced nutrient supply mode, tobacco plants lose their ability to actively regulate nutrient uptake through their root systems and can only passively accept fluctuating nutrient concentrations in the soil, ultimately resulting in imbalanced chemical composition within the tobacco leaves. In contrast, organic fertilizer application re-establishes the coordinated relationship between nutrient supply and plant demand. Organic fertilizers restore the decomposition and transformation functions of soil microorganisms through slow nutrient release, achieving good matching between nutrient supply rhythm and plant growth patterns (Yan et al., 2025). More importantly, the abundant bioactive substances in organic fertilizers (amino acids, humic acids, vitamins, etc.) can participate in tobacco leaf secondary metabolism processes and provide key precursor substances for aroma compound formation (Jiang et al., 2025). However, organic nitrogen application is not a case of “more is better,” as its obvious dose-dependent effects make determining the optimal organic–inorganic fertilizer ratio a key technical issue for achieving tobacco quality optimization.

Recent research on organic–inorganic fertilizer combinations has demonstrated that approximately 50% organic nitrogen substitution exhibits significant advantages in various crop production systems. In rice cultivation, Hou et al. (2024) found that compared with chemical fertilizer application alone, 50% organic nitrogen treatment significantly enhanced soil retention capacity for ammonium and nitrate nitrogen and optimized nitrogen fertilizer fate, increasing nitrogen use efficiency from 30.3% in chemical fertilizer application to 36.7%. Sun et al. (2022) showed that 50% organic nitrogen substitution significantly increased cereal yield by 14.39–15.58% while simultaneously improving rice palatability and nutritional quality, demonstrating the potential of this ratio in balancing yield and quality improvements. Chang et al. (2024) similarly found in citrus research that 50% organic nitrogen substitution of base chemical fertilizer achieved optimal effects in enhancing soil available nutrient content and citrus quality. This consistent performance across different crops highlights the uniqueness of the 50% organic nitrogen substitution. In tobacco research, this pattern has been further validated. Cao et al. (2012) demonstrated that organic nitrogen substitution for inorganic nitrogen improved the coordination of tobacco chemical composition and promoted beneficial mineral element accumulation, with 50% organic nitrogen substitution increasing tobacco economic output value by 44.42%, high-grade tobacco rate by 56.62%, and improving tobacco leaf chemical composition. Previous research integrating multi-site experimental data from China revealed that low organic nitrogen ratios (15–30%) primarily enhance yield, whereas medium ratios (50–60%) mainly improve the chemical quality of tobacco; however, the same study also found that the 50% substitution ratio is the optimal balance point for synergistically enhancing both (Zhu et al., 2024). This phenomenon indicates that different underlying mechanisms exist at different substitution ratios, particularly the soil microbial response mechanisms, which remain to be elucidated.

Organic fertilizer application establishes the foundation for microbial community reconstruction by improving soil physicochemical properties. Organic fertilizer input significantly regulates soil carbon-nitrogen ratio and pH, provides abundant carbon sources and other trace elements for microorganisms, and modifies soil structure, thereby promoting significant changes in microbial community diversity and structural composition (Bebber and Richards, 2022; Zhong et al., 2024). Organic fertilizer exhibits selective enrichment effects on functional microorganisms, such as significant enrichment of plant growth-promoting bacteria (PGPR) genera like Bacillus and Pseudomonas (Sabir et al., 2021). These key functional microbial groups enhance nutrient bioavailability and promote plant nutrient uptake through pathways including secreting plant hormones, dissolving mineral phosphorus, and synthesizing bioactive substances (Watzinger, 2015). Meanwhile, metagenomic analysis revealed that organic fertilizer treatment significantly increased the abundance of functional genes involved in key biogeochemical processes, including complex carbon compound degradation, ammonia oxidation (amoA), and biological nitrogen fixation (nifH), with significantly upregulated expression levels of related genes, thereby enhancing soil biochemical cycling capacity and achieving sustainable improvement of soil fertility (Shi et al., 2021; Sun et al., 2015). Organic fertilizer application ultimately achieves crop quality improvement by reshaping the structure and function of soil microbial communities, with microbial communities serving as a critical bridge connecting soil environment and crop quality. However, most current research on organic nitrogen application effects primarily focuses on apparent changes in agronomic traits and chemical composition, while lacking in-depth study of the microbiological mechanisms affecting crop quality. Particularly, how different organic nitrogen substitution ratios achieve differential effects on crop quality through regulating soil microbial communities, and the microbial ecological basis for why 50% substitution ratio exhibits optimal effects, remain important gaps in current research.

Based on the outstanding performance of 50% organic nitrogen substitution in previous studies, this study proposes the following hypothesis: 50% organic nitrogen substitution is the optimal scheme for optimizing tobacco yield and quality because this ratio can construct a soil microbial community that is the most structurally stable, functionally efficient, and best matched with plant demand. To test this hypothesis, we designed field experiments with three organic nitrogen substitution gradients centered around the hypothesized optimum. The 40% treatment was set to evaluate the effects of insufficient organic nitrogen substitution, the 60% treatment was designed to examine the consequences of exceeding the optimal ratio, and the 50% treatment served as the hypothesized optimum. This experimental design allows for a comprehensive assessment of the dose–response relationship and validates whether 50% represents the true optimum for tobacco production. The study systematically evaluated the effects of different treatments on tobacco agronomic traits, yield performance, chemical composition, and sensory quality, while employing 16S rRNA sequencing to comprehensively analyze changes in soil microbial community characteristics. This study aims to provide a scientific basis for understanding the microbiological foundations of organic fertilizer effects and holds important value for guiding precision fertilization in tobacco production.

## 2 Materials and methods

### 2.1 Experimental site

Field experiments were conducted at two locations, Huatian Village (HT) and Layuan Village (LY), in Xuntian Township, Xinning County, Shaoyang City, Hunan Province (26°47′08″N, 111°21′40″E) from April to August 2024. The region belongs to the mid-subtropical monsoon humid climate zone, with an annual average actual sunshine duration of 1465.6 h, annual average temperature of 17 °C, and annual average precipitation of 1331.1 mm. The experimental sites were located on flat terrain and employed a tobacco-rice rotation system.

### 2.2 Experimental design

The experiment adopted a randomized block design with 4 treatments at each location, replicated 3 times. Each treatment replication plot constituted one experimental unit, with each plot covering approximately 50 m^2^. The specific fertilization treatments were: no organic nitrogen application (CK), 40% organic nitrogen substitution (40% ON), 50% organic nitrogen substitution (50% ON), and 60% organic nitrogen substitution (60% ON). Total nitrogen application was equivalent across all treatments at 154 kg·ha^−1^, while phosphorus and potassium fertilizers were applied according to local standard fertilization practices (P₂O₅: 126 kg·ha^−1^, K₂O: 488 kg·ha^−1^). Commercial organic fertilizer was used as the organic nitrogen source (detailed composition shown in Table 1). The tobacco variety tested was “K326.”

**Table 1:** Composition and characteristics of the commercial organic fertilizer.

Composition	Value (%)
Organic matter	55.7
Total nutrients (N + P₂O₅ + K₂O)	7.7
Total nitrogen (N)	2.5
Total phosphorus (P₂O₅)	2.2
Total potassium (K₂O)	3.0
Moisture content	26

Raw material composition (%).

Tobacco stalk powder: 43.

Rapeseed cake: 20.

Rice husk biochar: 17.

Chicken manure: 11.

Rapeseed meal: 9.

### 2.3 Sample collection and measurement

This study measured tobacco plant height, stem diameter, and middle leaf area at three critical growth stages: rosette stage (35 days after transplanting), vigorous growth stage (70 days after transplanting), and maturity stage (110 days after transplanting) for subsequent analysis. Additional sample collection was conducted at the maturity stage. To comprehensively evaluate tobacco quality and soil environment, samples were collected in three categories: tobacco leaves, non-rhizosphere soil, and rhizosphere soil.

#### 2.3.1 Tobacco leaf collection

One to two uniformly developed middle leaves were collected from each plant as samples. The collected leaves were immediately placed in ice boxes and transported to the laboratory. Part of the tobacco leaves were heat-treated in a ventilated oven at 105 °C for 30 min, then dried at low temperature (60 °C) to constant weight. The dried leaf samples were ground and sieved for determination of tobacco leaf chemical composition. The remaining tobacco leaves were sent to partnering professional tobacco companies for sensory quality evaluation.

#### 2.3.2 Non-rhizosphere soil collection

Five non-rhizosphere soil sampling points were selected around each tobacco plant, 30–50 cm away from the main stem. Soil samples were collected using soil augers at 0–20 cm depth, then thoroughly mixed from these five sampling points to form a composite sample. Stones and plant debris were removed from the composite samples, which were then labeled and packed in sterile sealed bags. These samples were used for determination of soil total nitrogen, total carbon, and acid-hydrolyzable nitrogen.

#### 2.3.3 Rhizosphere soil collection

Soil closely adhered to tobacco plant roots from each treatment was collected and mixed to form one composite sample per treatment. After removing stones and plant debris, soil samples were subdivided. One portion was immediately frozen and stored at −80 °C for subsequent soil bacterial microbiome analysis. Another portion was stored at 4 °C for determination of soil lignin content, microbial residue carbon content, and phospholipid fatty acid (PLFA) indicators.

### 2.4 Leaf sample determination and sensory quality evaluation

Mineral element content determination: Potassium was determined using flame photometry following the method of Lu et al. (2017); chloride was measured using the nitric acid-glacial acetic acid extraction-silver ion titration method established by Gilliam (1971), with quantitative analysis conducted using a chloride analyzer.

*Nitrogen content determination*: Total nitrogen content in leaves was determined using the Kjeldahl method (Bremner, 1965).

*Carbohydrate content determination*: Both starch content and total sugar content were determined using the anthrone-sulfuric acid colorimetric method, following the method established by Dreywood (1946), with absorbance measured at 620 nm wavelength using a spectrophotometer.

*Nicotine content determination*: Nicotine content was determined using the distillation-spectrophotometry method, where leaf samples were subjected to distillation treatment, the distillate was collected with sulfuric acid solution, then diluted to volume and measured for characteristic absorption peak absorbance using UV spectrophotometer (Al-Tamrah, 1999).

*Sensory quality evaluation*: Tobacco sensory quality was evaluated by professional sensory panelists from cooperative tobacco companies according to industry standards, with comprehensive scoring conducted for traits such as aroma quality, aroma intensity, off-flavors, and irritation of each treatment sample.

### 2.5 Soil sample determination and DNA extraction

Soil total nitrogen and acid-hydrolyzable nitrogen contents were both determined using the Kjeldahl method, following the method established by Bremner (1996) for sample digestion, distillation, and titration. Soil organic carbon content was determined using the Walkley-Black wet oxidation method (Nelson and Sommers, 1996). Soil phospholipid fatty acids (PLFAs) were extracted and analyzed using the method established by Brant et al. (2006), with microbial community composition quantitatively analyzed through gas chromatography–mass spectrometry. Soil microbial residue carbon was determined using the chloroform fumigation-extraction method, following the method of Vance et al. (1987). Soil lignin-derived phenolic compounds were extracted using the nitrobenzene oxidation method, following the method established by Xiao et al. (2001), with lignin monomer composition and content analyzed using high-performance liquid chromatography.

Soil DNA extraction was performed using the E.Z.N.A.® soil DNA kit (Omega Bio-tek, Norcross, GA, U.S.) according to the instructions of manufacturer. The quality of extracted genomic DNA was assessed using 1% agarose gel electrophoresis, and DNA concentration and purity were measured using NanoDrop2000 (Thermo Scientific, USA).

### 2.6 PCR amplification and Illumina sequencing

Using the extracted DNA as template, the V3-V4 hypervariable region of the 16S rRNA was amplified by PCR using barcode-tagged upstream primer 338F (5’-ACTCCTACGGGAGGCAGCAG-3′) and downstream primer 806R (5’-GGACTACHVGGGTWTCTAAT-3′) (Liu et al., 2016). The PCR reaction mixture consisted of: 4 μL 5 × TransStart FastPfu buffer, 2 μL 2.5 mM dNTPs, 0.8 μL upstream primer (5 μM), 0.8 μL downstream primer (5 μM), 0.4 μL TransStart FastPfu DNA polymerase, 10 ng template DNA, and ddH₂O to a final volume of 20 μL. The amplification program was as follows: initial denaturation at 95 °C for 3 min, followed by 27 cycles of denaturation at 95 °C for 30s, annealing at 55 °C for 30s, and extension at 72 °C for 30s, then final extension at 72 °C for 10 min, and storage at 4 °C (PCR instrument: ABI GeneAmp® 9700).

PCR products were recovered from 2% agarose gel and purified using a DNA gel recovery purification kit (PCR Clean-Up Kit, YuHua, China), and the recovered products were quantified using Qubit 4.0 (Thermo Fisher Scientific, USA). Library construction was performed on the purified PCR products using the NEXTFLEX Rapid DNA-Seq Kit: (1) adapter ligation; (2) magnetic bead selection to remove adapter dimers; (3) PCR amplification for library template enrichment; (4) magnetic bead recovery of PCR products to obtain the final library. Sequencing was performed on the Illumina Nextseq2000 platform (Shanghai Majorbio Bio-Pharm Technology Co., Ltd.). Raw data were uploaded to the NCBI SRA database (Accession Number: PRJNA1327578).

### 2.7 Bioinformatics analysis

Raw paired-end sequencing data were quality controlled using fastp (v0.19.6) with quality threshold set at 20, sliding window size of 50 bp, removing low-quality bases and reads containing N, and filtering sequences shorter than 50 bp (Chen et al., 2018). Quality-controlled paired-end reads were merged using FLASH (v1.2.11) with minimum overlap length set at 10 bp and maximum mismatch rate in overlap region of 0.2.

Samples were classified and oriented according to barcode and primer sequences, with exact barcode matching and allowing maximum 2 mismatches in primers (Magoč and Salzberg, 2011). High-quality sequences were clustered into OTUs using UPARSE (v7.1) with 97% similarity, and chimeric sequences were removed (Edgar, 2013; Stackebrandt and Goebel, 1994). To eliminate the impact of sequencing depth differences on diversity analysis, sequence counts from all samples were normalized to 20,000, achieving an average coverage of 99.09% after rarefaction. Taxonomic annotation was performed using RDP classifier (v2.11) based on the Silva 16S rRNA database (v138) with confidence threshold set at 70%, and community composition at various taxonomic levels was determined (Wang et al., 2007).

### 2.8 Statistical analysis

Microbiome data analysis was conducted on the Majorbio Bioinformatics Cloud Platform^1^ as follows: Alpha diversity indices including Chao1 and Shannon indices were calculated using mothur software (Schloss et al., 2009), and Wilcoxon rank-sum test was employed for between-group differences in alpha diversity analysis. Principal coordinates analysis (PCoA) based on Bray-Curtis distance algorithm was used to examine the similarity of microbial community structure between samples, combined with PERMANOVA non-parametric test to analyze whether microbial community structure differences between sample groups were significant. Kruskal-Wallis test was used to analyze between-group differences in species abundance at the genus level. LEfSe analysis (Linear discriminant analysis Effect Size, LDA > 3, *p* < 0.05) was employed to identify bacterial groups with significantly different abundances from phylum to genus level between different groups (Segata et al., 2011). Distance-based redundancy analysis (db-RDA) was used to investigate the effects of soil bacterial communities on tobacco leaf chemical composition. Correlation network analysis was performed on selected species based on Spearman correlation |r| > 0.6, *p* < 0.05 (Barberán et al., 2012). FAPROTAX database was used for functional prediction analysis of soil carbon and nitrogen cycling based on 16S rRNA data (Louca et al., 2016).

Data related to soil physicochemical properties, tobacco growth, and yield were statistically analyzed using R software (version 4.4.2), with one-way analysis of variance used to test differences between treatments, Duncan’s method for multiple comparisons, and significance level set at *p* < 0.05. Figures and tables were created using R language “ggplot2” package and Microsoft Excel.

## 3 Results

### 3.1 Tobacco agronomic traits and yield performance

**Figure 1:**
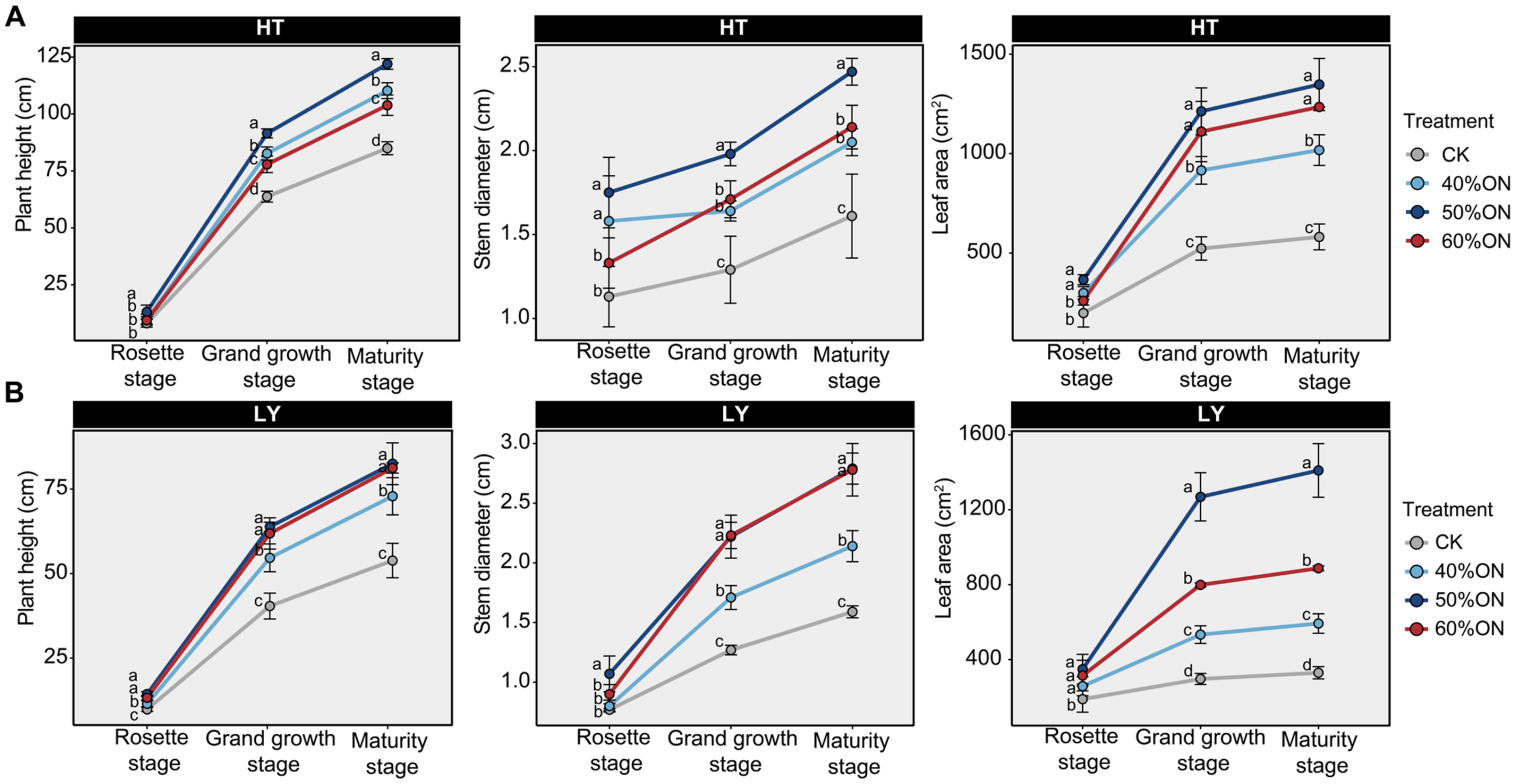
Effects of different organic nitrogen treatments on tobacco agronomic traits during three growth stages. **(A)** HT experimental site, **(B)** LY experimental site. Different letters indicate statistically significant differences (*p* < 0.05).

Tobacco agronomic trait results showed that at the HT site, the 50% ON treatment exhibited the largest plant height, stem diameter, and leaf area during both vigorous growth and maturity stages. The 40% ON and 60% ON treatments were smaller than the 50% ON treatment, but all three organic nitrogen treatments showed agronomic traits significantly higher than the CK (Figure 1A). At the LY site, the 50% ON and 60% ON treatments showed no significant differences in tobacco plant height and stem diameter during vigorous growth and maturity stages, both significantly greater than 40% ON and greater than the CK. Regarding leaf area, the 50% ON treatment demonstrated the largest tobacco leaf area, following the trend of 50% ON > 60% ON > 40% ON > CK (Figure 1B).

**Figure 2:**
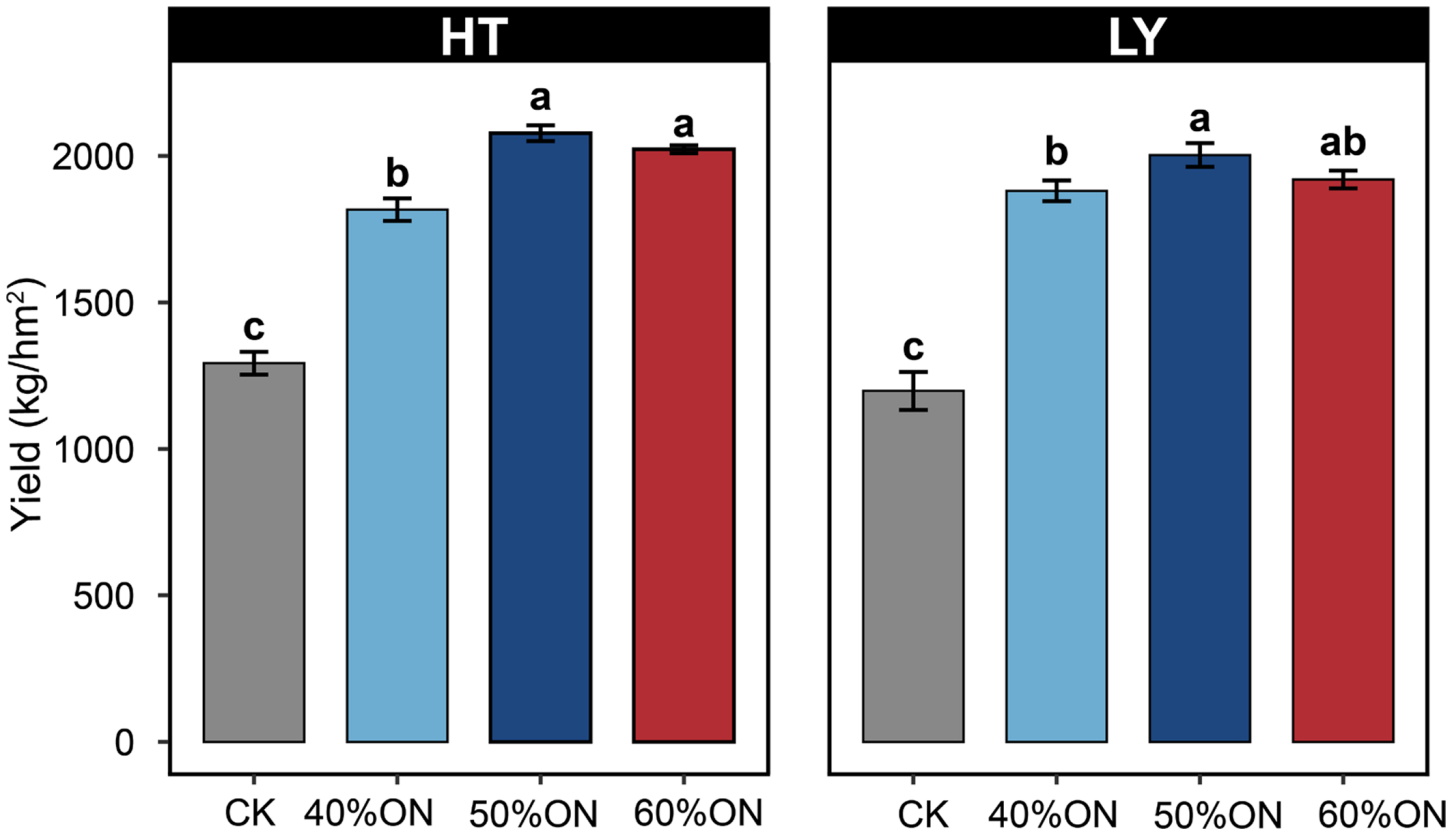
Effects of different organic nitrogen treatments on tobacco yield at two experimental sites. Different letters indicate statistically significant differences (*p* < 0.05).

Different organic nitrogen substitution ratios significantly affected tobacco yield at both experimental sites. Compared with the CK, organic nitrogen application significantly increased tobacco yield. The 40% ON, 50% ON, and 60% ON treatments increased yield by 42.9, 63.4, and 58.2% at the HT site, and by 59.2, 67.8, and 62.4% at the LY site, respectively. Both sites exhibited similar trends: the 50% ON treatment achieved the highest yield (HT: 2077.38 kg/ha, LY: 2002.98 kg/ha), but showed no significant difference from the 60% ON treatment while being significantly higher than the 40% ON treatment (Figure 2).

### 3.2 Tobacco leaf chemical composition and sensory quality

**Figure 3:**
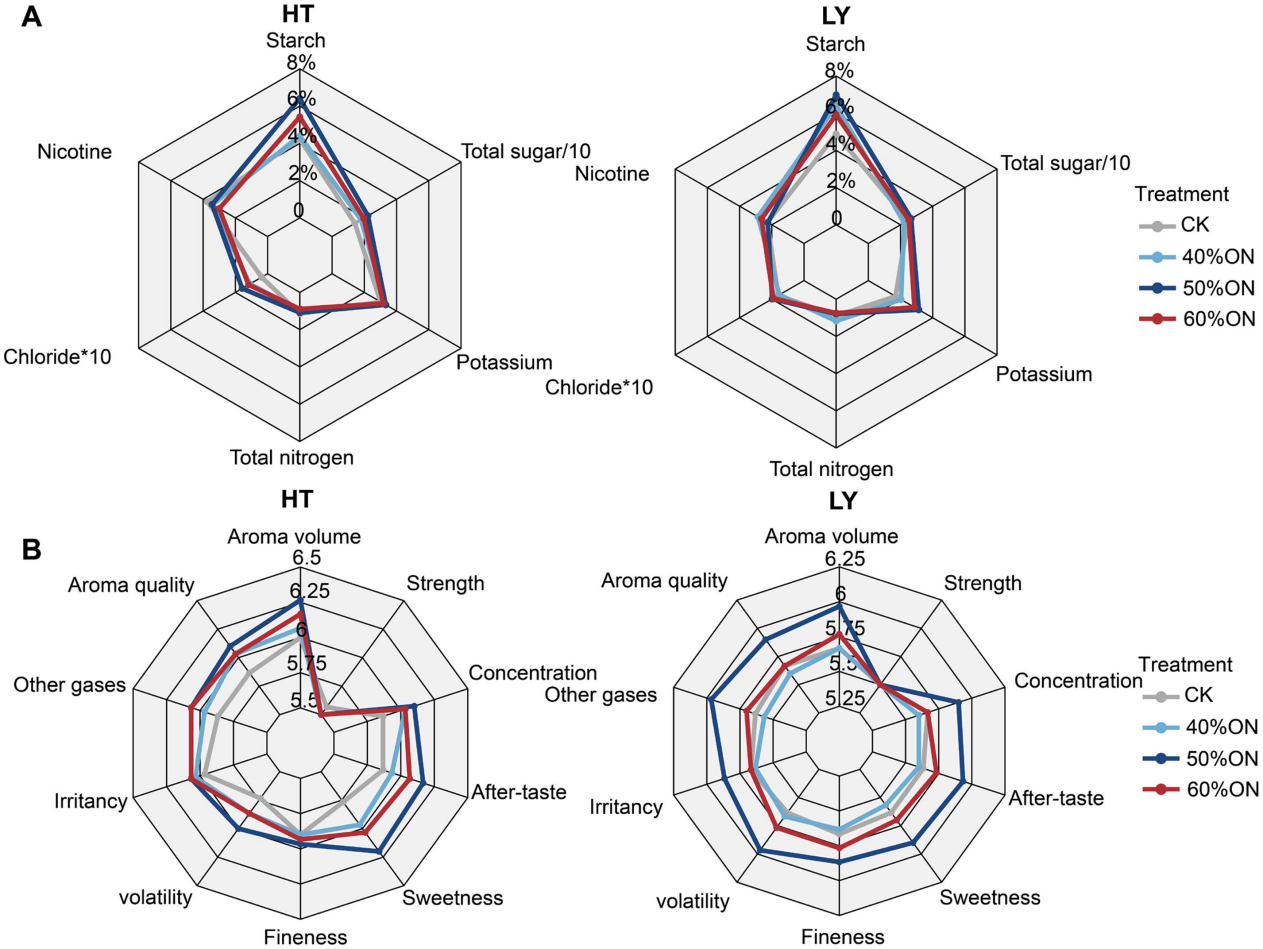
Effects of different organic nitrogen treatments on **(A)** tobacco leaf chemical composition and **(B)** sensory quality at two experimental sites. Percentage values in radar chart A represent the content of each chemical component in tobacco leaves. Values in radar chart B represent the scores of sensory quality indicators.

The application of organic nitrogen altered the chemical composition of tobacco leaves at both sites. Specifically, it increased the content of starch, potassium, total sugar, and chloride, with the treatments ranking as follows: 50%ON > 60%ON > 40%ON > CK (Figure 3A). Conversely, organic nitrogen application at the HT site significantly decreased both nicotine and total nitrogen content, with the 60%ON treatment demonstrating the greatest reduction. At the LY site, the 40%ON treatment increased nicotine and total nitrogen content relative to the CK. In contrast, the 50%ON treatment reduced both, while the 60%ON treatment increased nicotine but decreased total nitrogen content (Figure 3A).

In terms of sensory quality, at the HT site, the application of organic nitrogen improved the sensory quality of tobacco leaves; the overall sensory quality of the 50%ON and 60%ON treatments was similar, and both were superior to the 40%ON treatment. At the LY site, there was no significant difference in the sensory quality of tobacco leaves between the CK and the 40%ON treatment; the sensory quality of the 50%ON and 60%ON treatments was significantly improved, with the 50%ON treatment exhibiting the best overall sensory quality (Figure 3B).

### 3.3 Soil physicochemical properties and microbial activity

In terms of soil physicochemical properties, both sites showed that organic nitrogen treatments significantly affected the soil nutrient status. The soil total nitrogen content at both sites was significantly higher in the CK and 60%ON treatments than in the 40%ON and 50%ON treatments; the total carbon content was highest in the 60%ON treatment at the HT site, with no significant difference from the 50%ON treatment, and highest in the 50%ON treatment at the LY site, with no significant difference from the 60%ON treatment, but at both sites, the 50%ON and 60%ON treatments were significantly higher than the CK and 40%ON treatments. The acid-hydrolyzable nitrogen content at both sites was significantly higher in the 50%ON treatment than in the other treatments, the 60%ON treatment was significantly higher than the CK, while there was no significant difference between the 40%ON and CK. The lignin content at both sites showed a trend of 50%ON > 60%ON > 40%ON > CK, but there were no significant differences among the treatments (Table 2).

**Table 2:** Effects of different organic nitrogen treatments on soil physicochemical properties at two experimental sites.

Site	Treatment	Total nitrogen (g/kg)	Total carbon (g/kg)	Acid-hydrolyzable nitrogen (g/kg)	Lignin (mg/kg)	PLFAs (nmol/g)
HT	CK	1.68 ± 0.09a	14.89 ± 0.60b	1.26 ± 0.38c	463.26 ± 20.17a	16.67 ± 1.04b
	40%ON	1.66 ± 0.04a	15.21 ± 0.44b	1.30 ± 0.19bc	477.22 ± 12.59a	17.79 ± 1.24ab
	50%ON	1.49 ± 0.09b	15.72 ± 1.15ab	1.43 ± 0.17a	484.23 ± 37.80a	19.39 ± 1.52a
	60%ON	1.43 ± 0.11b	17.04 ± 0.84a	1.34 ± 0.25b	477.84 ± 19.25a	19.96 ± 0.98a
LY	CK	2.01 ± 0.13a	17.00 ± 0.29b	1.41 ± 0.02c	631.75 ± 7.43a	19.22 ± 0.88a
	40%ON	1.83 ± 0.09ab	17.13 ± 0.53b	1.47 ± 0.05c	666.15 ± 26.30a	19.49 ± 6.6a
	50%ON	1.74 ± 0.04b	18.08 ± 0.55a	1.70 ± 0.04a	695.38 ± 107.66a	19.86 ± 1.63a
	60%ON	1.80 ± 0.14b	17.42 ± 0.41ab	1.56 ± 0.06b	691.30 ± 4.09a	20.9 ± 3.88a

Different letters indicate statistically significant differences (*p* < 0.05).

Regarding soil microbial activity, at the HT site, the PLFAs content was significantly higher in the 50%ON treatment than in the other treatments, the 60%ON was significantly higher than CK, and there was no significant difference between 40%ON and CK. At the LY site, there were no significant differences in PLFAs content among the treatments, but it showed a trend of 60%ON > 50%ON > 40%ON > CK (Table 2).

The bacterial, fungal, and total microbial residue carbon all showed a decreasing trend of 60%ON > 50%ON > 40%ON > CK with the reduction of organic nitrogen content: at the HT site, all three organic nitrogen treatments were significantly higher than the CK; at the LY site, there were significant differences among the 60%ON, 50%ON, and 40%ON treatments, but no significant difference was found between the 40%ON treatment and CK (Supplementary Figure S1).

### 3.4 Soil microbial community diversity and structure

**Figure 4:**
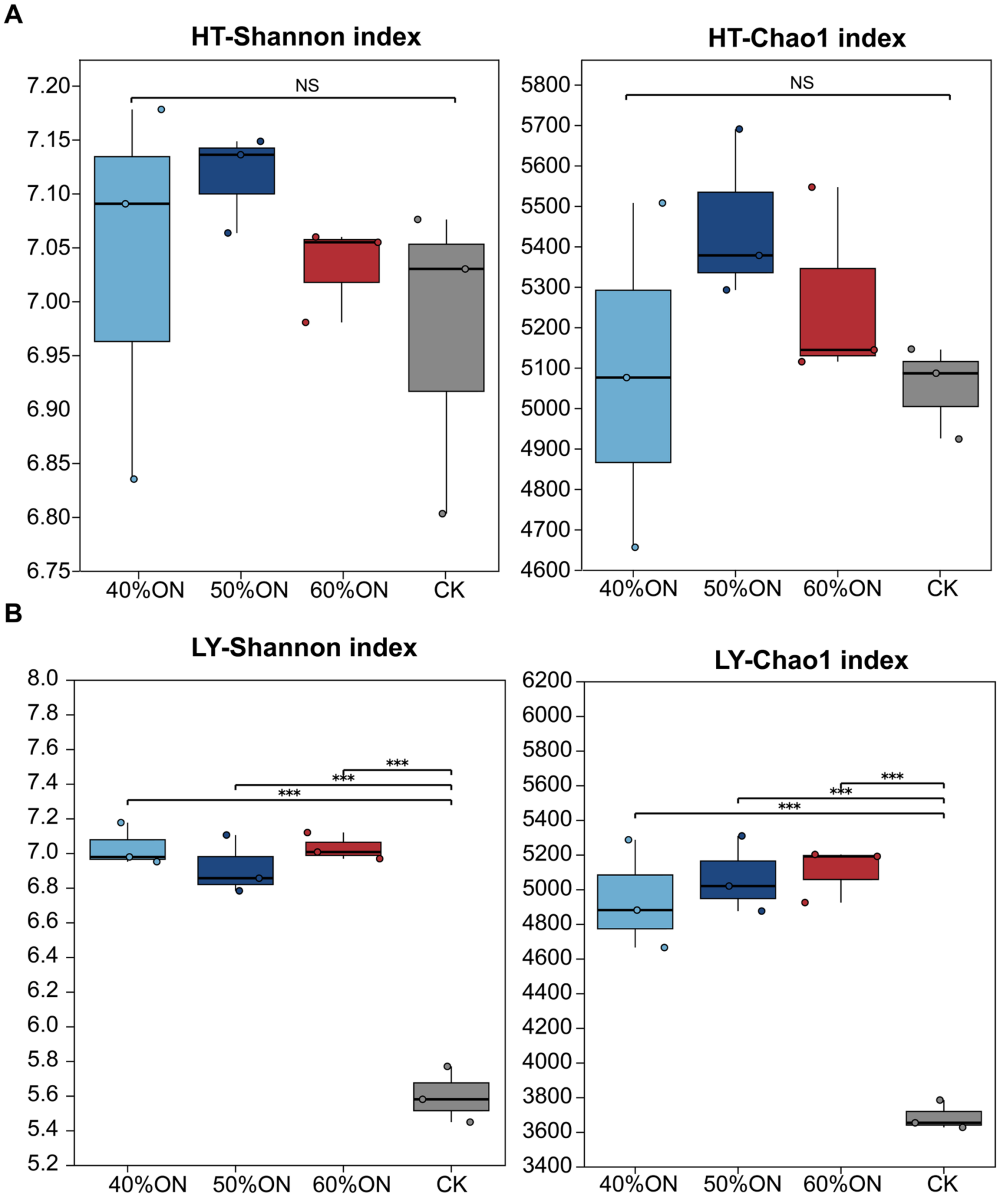
Effects of different organic nitrogen treatments on soil bacterial Shannon and Chao1 indices. **(A)** HT experimental site, **(B)** LY experimental site. Significance thresholds between treatments are * *p* < 0.05, ** *p* < 0.01, *** *p* < 0.001 and NS means non-significant.

The application of organic nitrogen increased soil microbial diversity at both experimental sites. At the HT site, the Shannon and Chao1 indices for the 40%ON, 50%ON, and 60%ON treatments were higher than those for the CK (Shannon index: 50%ON > 40%ON > 60%ON; Chao1 index: 50%ON > 60%ON > 40%ON), although no significant differences were observed among the four treatments (Figure 4A). At the LY site, the Shannon and Chao1 indices of the 40%ON, 50%ON, and 60%ON treatments were significantly higher than those of the CK (Shannon index: 60%ON > 40%ON > 50%ON; Chao1 index: 60%ON > 50%ON > 40%ON), but no significant differences were found among these three organic nitrogen treatments (Figure 4B).

**Figure 5:**
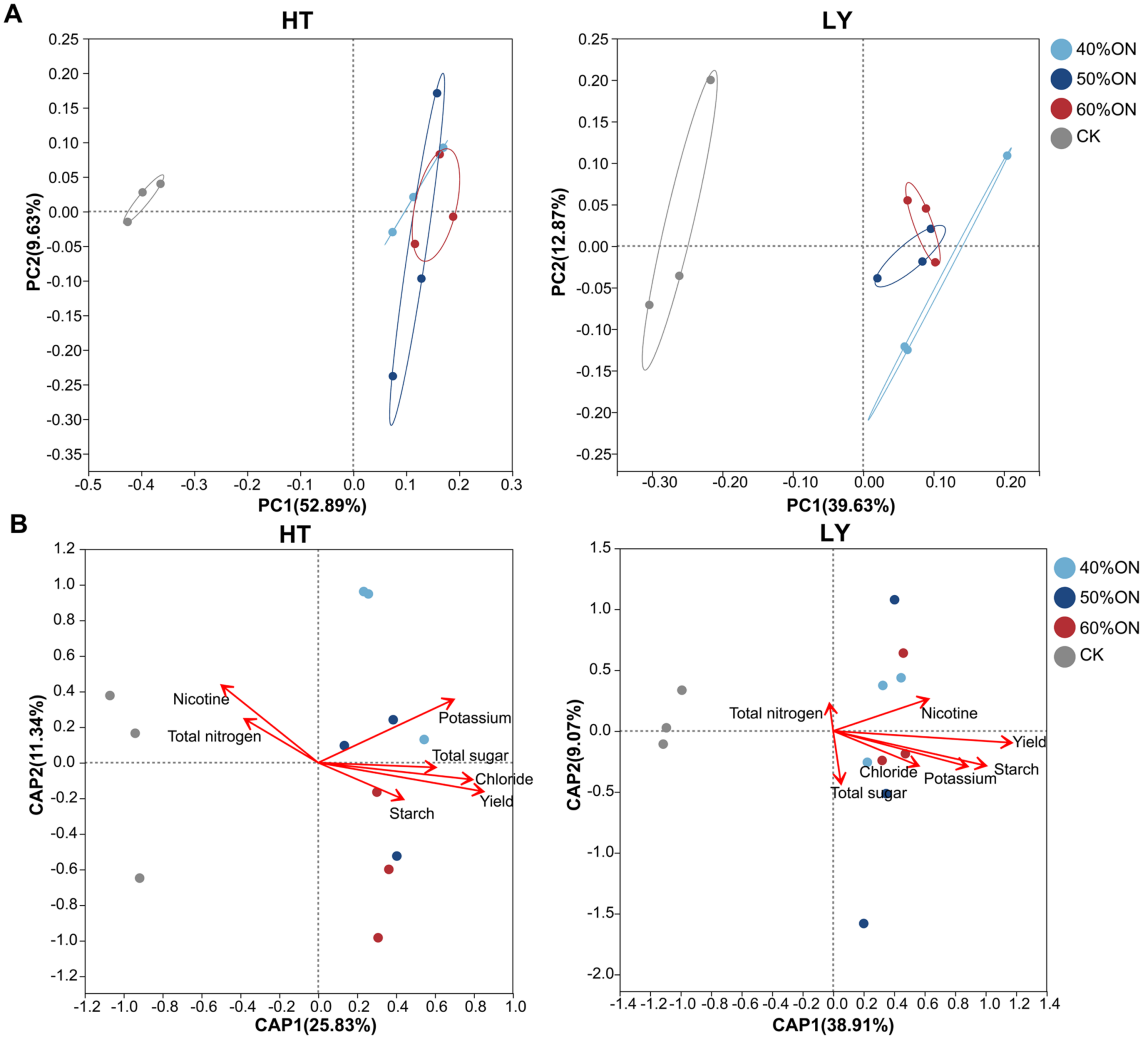
Effects of different organic nitrogen treatments on soil bacterial community structure and its relationship with leaf chemical composition at two experimental sites. **(A)** Principal coordinate analysis (PCoA), **(B)** distance-based redundancy analysis (db-RDA).

PCoA at the OTU level revealed that the application of organic nitrogen significantly influenced the soil microbial community structure at both sites. At the HT site, the first two principal coordinate axes collectively explained 52.60% of the community variation (PC1: 39.63%, PC2: 12.97%). The three organic nitrogen treatments were clearly separated from the CK; furthermore, the 40%ON treatment was slightly separated from the 50%ON and 60%ON treatments, while the latter two clustered together (Figure 5A). At the LY site, the first two principal coordinate axes explained 59.52% of the community variation (PC1: 52.89%, PC2: 6.63%). A clear separation was observed between the three organic nitrogen treatments and the CK, whereas the three organic nitrogen treatments themselves did not separate from one another (Figure 5B).

**Figure 6:**
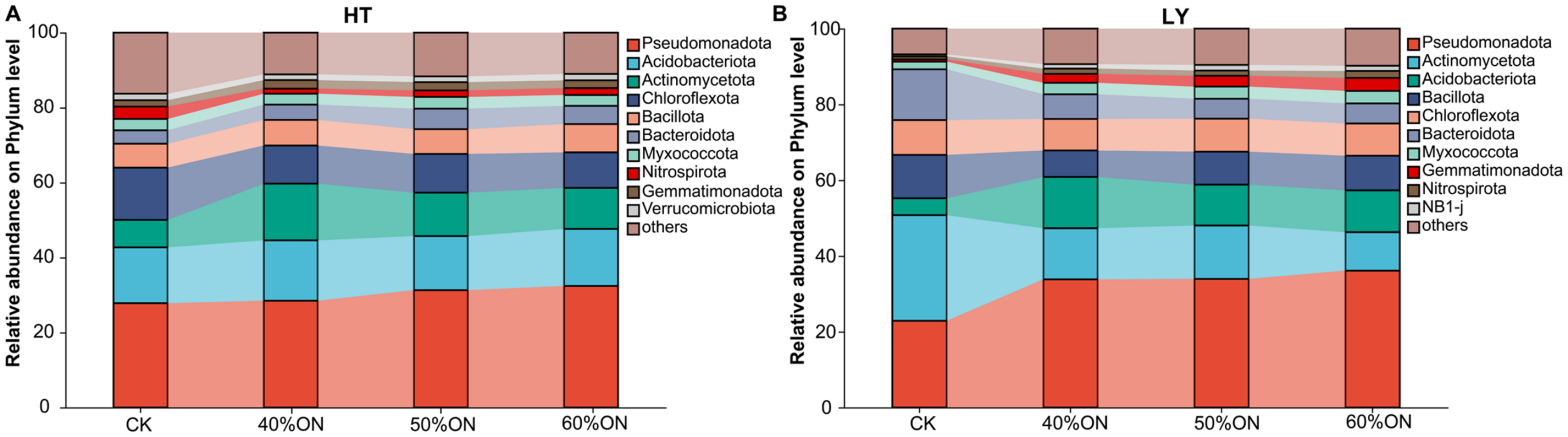
Effects of different organic nitrogen treatments on soil bacterial community composition at the phylum level. **(A)** HT experimental site, **(B)** LY experimental site.

At the HT site, the bacterial communities under the different fertilization treatments were dominated by the phyla Pseudomonadota, Acidobacteriota, Actinomycetota, Chloroflexota, Bacillota, Bacteroidota, Myxococcota, Nitrospirota, and Gemmatimonadota. Compared to the CK, all three organic nitrogen treatments exhibited a notable increase in the relative abundance of Pseudomonadota, Actinomycetota, and Bacteroidota, while the proportions of Chloroflexota and Nitrospirota decreased. Among the three organic nitrogen treatments, the 50%ON and 60%ON treatments showed similar microbial compositions; relative to the 40%ON treatment, they featured a slight increase in the proportions of Pseudomonadota and Bacteroidota, alongside a decrease in Actinomycetota (Figure 6A). The composition of the bacterial community at the LY site was highly similar to that at the HT site. Here, the three organic nitrogen treatments shared a similar community structure, which differed considerably from that of the CK; specifically, compared to the CK, these treatments showed a marked increase in the relative abundance of Pseudomonadota, Actinomycetota, Myxococcota, and Gemmatimonadota, contrasted with a decrease in Acidobacteriota, Bacillota, and Bacteroidota. Within the organic nitrogen treatments, the microbial compositions of the 50%ON and 60%ON treatments were again similar; compared to the 40%ON treatment, both showed decreased abundances of Actinomycetota and Bacteroidota but increased abundances of Pseudomonadota and Bacillota. Furthermore, when comparing the 50%ON and 60%ON treatments directly, the 50%ON treatment had a higher proportion of Acidobacteriota and a lower proportion of Pseudomonadota (Figure 6B).

At the HT site, distinct indicator taxa (LDA score ≥ 3) were identified for each treatment. The 40%ON treatment was characterized by taxa related to Actinomycetota, the family Rhodobacteraceae, groups within Thermomicrobia, and the order Gaiellales; the key indicator for the 50%ON treatment was taxa associated with the phylum Bacteroidota; and biomarkers for the 60%ON treatment included the genera Sphingobacterium, Sphingomonas, and Steroidobacter. In the CK, characteristic taxa included Gammaproteobacteria, the order Burkholderiales, the family Pseudomonadaceae, and the genus Pseudomonas (all within Proteobacteria), as well as unclassified species from the phyla Nitrospirota and Sva0485 (Supplementary Figure S2). At the LY site, biomarkers for the 40%ON treatment primarily included the order Burkholderiales, the family Comamonadaceae, the genus Acidibacter, the order Azospirillales, the genus Steroidobacter, and several unclassified microorganisms. Indicator taxa for the 50%ON treatment mainly comprised the order Polyangiales, the order PLTA13, the class Polyangia, the family Blrii41, along with unclassified species within the class OLB14 and the order PLTA13. Biomarkers for the 60%ON treatment included the phylum Gemmatimonadota and its associated taxa (the order Gemmatimonadales, family Gemmatimonadaceae, and class Gemmatimonadia), the family Flavobacteriaceae, taxa within the class Nitrospiria (the order Nitrospirales, family Nitrospiraceae, and genus Nitrospira), and the class Chloroflexia. In the CK, the significantly enriched microbial taxa comprised the phylum Actinomycetota and its related groups (the order Micrococcales, order Propionibacteriales, family Nocardiaceae, and family Intrasporangiaceae), in addition to the genera Pediococcus-Algicoccus, the family Membranilaceae, the order Cytophagales, the genus Maribacter, and the genus Sphingomonas (Supplementary Figure S3).

The db-RDA analysis revealed distinct differences in the tobacco leaf quality traits associated with the microbial communities of the CK and organic nitrogen treatments. At the HT site, the microbial community of the CK was primarily associated with nicotine and total nitrogen, while at the LY site, it was mainly associated with total nitrogen. In contrast, the microbial communities of the organic nitrogen treatments (40%ON, 50%ON, and 60%ON) at both sites correlated with yield. Meanwhile, at the HT site, they were associated with potassium, total sugar, starch, and chloride. At the LY site, the associated traits were nicotine, chloride, potassium, starch, and total sugar (Figures 5C,D).

### 3.5 Soil microbial community function and network characteristics

**Figure 7:**
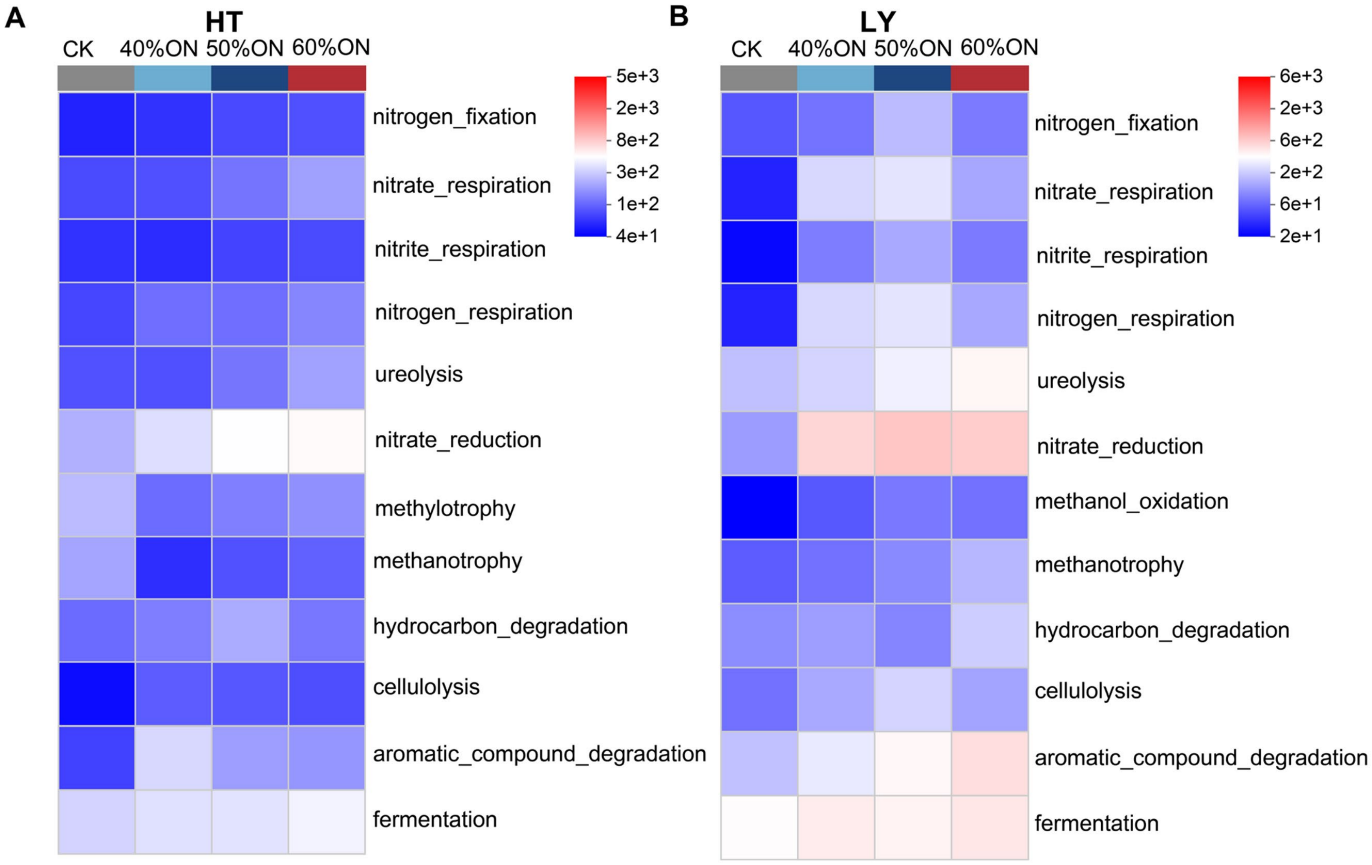
Effects of different organic nitrogen treatments on carbon and nitrogen cycling functions of soil microbial communities predicted by FAPROTAX analysis. **(A)** HT experimental site, **(B)** LY experimental site.

Functional prediction analysis based on the FAPROTAX database revealed that organic nitrogen treatments significantly altered the functional structure of the soil microbial community, with a functional heatmap clearly illustrating the differentiated patterns of carbon and nitrogen cycling functions among treatments. At the HT site, organic nitrogen treatments significantly enhanced functions related to nitrogen cycling, including nitrogen fixation, nitrate reduction, nitrite reduction, nitrogen respiration, and ureolysis; the functional abundance showed a dose-dependent increase with the proportion of organic nitrogen (40%ON < 50%ON < 60%ON). Regarding carbon cycling, these treatments notably promoted hydrocarbon degradation, cellulolysis, aromatic compound degradation, and fermentation, whereas the CK exhibited higher abundances in methylotrophy and methanotrophy. The 50%ON treatment maintained high and stable activity across all four mentioned carbon cycling functions (hydrocarbon degradation, cellulolysis, aromatic compound degradation, and fermentation), demonstrating the most balanced performance (Figure 7A). At the LY site, organic nitrogen treatments also significantly enhanced nitrogen cycling functions, with the 50%ON treatment showing the highest predicted functional abundance. For carbon cycling, the treatments promoted methane oxidation, hydrocarbon degradation, cellulolysis, aromatic compound degradation, and fermentation; the 60%ON treatment had the highest abundance in all these functions except for cellulolysis (Figure 7B).

Analysis of the topological properties of soil bacterial co-occurrence networks constructed for each treatment indicated that organic nitrogen application increased the complexity of the microbial network at both sites. At the HT site, the number of edges in the 40%ON, 50%ON, and 60%ON treatments increased by 17.55, 21.81, and 5.85%, respectively; at the LY site, the increases were 6.49, 22.86, and 5.45%. Notably, the 50%ON treatment exhibited the most complex network at both sites, reaching an average degree of 18.694 (HT) and 18.92 (LY) and a network density of 0.389 (HT) and 0.386 (LY), respectively. Regarding microbial interaction patterns, the organic nitrogen treatments significantly altered the association patterns within the bacterial networks at both sites. The 40%ON and 50%ON treatments showed an increase in negative correlations, while the 60%ON treatment showed a decrease; the 50%ON treatment exhibited the highest number of negative edges at both sites. Furthermore, the modularity index for all organic nitrogen treatments decreased significantly at both sites; the reductions at the HT site were 10.78, 28.10, and 1.14%, and at the LY site were 15.73, 21.37, and 2.39%, respectively, with the 50%ON treatment again showing the most pronounced decrease in both cases (Tables 3, 4; Supplementary Figures S4, S5).

**Table 3:** Effects of different organic nitrogen treatments on topological properties of soil bacterial networks at the HT experimental site.

HT	CK	40%ON	50%ON	60%ON
Number of nodes	48	50	49	49
Number of edges	376	442	458	398
Positive edges	205	226	232	246
Negative edges	171	216	226	152
Average degree	15.667	17.68	18.694	16.25
Network density	0.333	0.361	0.389	0.338
Modularity	0.612	0.546	0.44	0.605

**Table 4:** Effects of different organic nitrogen treatments on topological properties of soil bacterial networks at the LY experimental site.

LY	CK	40%ON	50%ON	60%ON
Number of nodes	48	50	50	49
Number of edges	385	410	473	406
Positive edges	217	220	245	223
Negative edges	168	190	228	183
Average degree	16.042	16.4	18.92	16.571
Network density	0.341	0.335	0.386	0.345
Modularity	0.585	0.493	0.46	0.571

## 4 Discussion

### 4.1 Verification of the optimality of 50% organic nitrogen substitution

This study verified through comprehensive evaluation of agronomic traits, yield performance, chemical composition, and sensory quality that organic nitrogen application significantly improved the overall performance of tobacco. Compared to the control group, all three organic nitrogen treatments showed obvious yield increase effects and quality improvement (Figures 1–3). The core finding of this study is that 50% organic nitrogen substitution (50% ON) represents the optimal scheme for achieving high yield and quality, as it achieved the highest yield levels at both experimental sites, with increases of 63.4 and 67.8%, respectively, compared to the control group (Figure 2). The 50% ON treatment not only performed optimally in terms of yield but also achieved significant improvements in the balance of chemical components and sensory quality. This treatment increased beneficial components such as potassium and total sugars while effectively controlling nicotine and total nitrogen content, promoting coordinated development of tobacco leaf quality (Figure 2). However, the advantages of 50% ON over 60% ON were not always statistically significant, which conforms to the law of diminishing marginal returns of fertilizer input—when the substitution rate exceeds 50%, continuing to increase the organic nitrogen ratio has very limited effect on yield improvement.

From the perspective of soil nitrogen supply, although the 60% ON treatment had higher total soil nitrogen content, the 50% ON treatment showed significantly higher acid-hydrolyzable nitrogen content than other treatments at both experimental sites (Table 2). Acid-hydrolyzable nitrogen represents the active nitrogen pool directly available to plants in soil, and its content directly determines the effectiveness of soil nitrogen supply (Chen et al., 2023; Wu et al., 2019). This indicates that soil nitrogen in the 60% ON treatment mainly existed in stable organic forms that are difficult for plants to utilize in the short term. Its acid-hydrolyzable nitrogen content was actually lower than that of the 50% ON treatment, reflecting that excessive organic matter input adversely affected soil nitrogen mineralization processes (Peng et al., 2023). Meanwhile, organic nitrogen costs more than chemical fertilizers, and higher proportions mean higher investment costs.

Therefore, from the perspectives of resource utilization efficiency and ecological sustainability, 50% ON has greater advantages. Changes in soil microbial-related indicators further revealed the biological basis for the advantages of the 50% ON treatment. Indicators including lignin content, total phospholipid fatty acids (PLFA), and microbial residue carbon all showed significantly higher levels in the 50% ON treatment compared to other treatments (Table 2; Supplementary Figure S1). The significant increase in PLFA content reflected increased microbial biomass and enhanced community activity (Watzinger, 2015). The enrichment of microbial residue carbon indicated more active microbial turnover, facilitating organic matter decomposition and transformation, improving nutrient bioavailability, while expanding stable carbon pools and enhancing soil carbon sequestration potential (Liu et al., 2024). As a representative of complex organic compounds, lignin content changes reflect the accumulation status of recalcitrant organic matter in soil. The relatively high lignin content in the 50% ON treatment indicated that the organic matter input in this treatment contained more structural carbon components, while microorganisms preferentially decomposed easily degradable components, achieving a good balance between organic matter input and microbial decomposition (Thevenot et al., 2010). These systematic changes in microbial-related indicators demonstrated that differences in organic nitrogen substitution ratios not only affected soil chemical properties, but more importantly, altered the compositional structure and functional characteristics of soil microbial communities.

### 4.2 Restructuring effects of organic nitrogen substitution on soil microbial community structure

Organic fertilizer application had profound restructuring effects on soil microbial communities, with these effects mainly manifested in significant increases in diversity and changes in community structure. At both experimental sites, all organic nitrogen treatments resulted in higher Shannon and Chao1 indices than the control group, indicating that organic nitrogen application effectively enhances the richness and diversity of soil microbial communities (Figure 4).

The application of organic fertilizer provides abundant carbon substrates, which may initially promote the rapid growth of certain copiotrophic microorganisms. Over time, diversified organic matter decomposition processes gradually unfold, with different decomposition stages providing suitable habitats for microorganisms occupying different ecological niches. This expansion of ecological niches becomes the primary driver for increased microbial community richness, ultimately leading to significant enhancement of overall diversity (Hartmann et al., 2015).

PCoA results further confirmed the restructuring effect of organic nitrogen on microbial community structure, revealing a clear separation between the organic nitrogen treatments and the control group at both sites (Figure 5A). Notably, community structure differences among different organic nitrogen substitution ratios were relatively small. Particularly at the LY site, the three organic nitrogen treatments showed essentially no separation, indicating that once organic nitrogen is applied, the primary trajectory of community change is consistent. The differences in substitution ratios mainly affect the magnitude rather than the direction of the change. This is consistent with Wang et al. (2024), who observed in rapeseed soil microbial communities that organic nitrogen treatments were clearly separated from chemical fertilizer, but there was some degree of overlap between organic nitrogen treatments.

Db-RDA analysis revealed significant associations between soil microbial community structure and both the chemical composition and yield of tobacco leaves. The microbial communities in the control group were mainly positively correlated with nicotine and total nitrogen, while those in the organic nitrogen treatments were significantly associated with yield, potassium, total sugars, starch, and chloride content, showing clear differentiation patterns among treatments (Figure 5B). The chemical composition of tobacco leaves is a critical indicator for evaluating their quality. Research has shown that sugar and potassium content are closely related to aroma and taste quality (Du et al., 2011; Shen et al., 2019). Although nicotine can neutralize acidic substances from sugar combustion, excessive amounts negatively affect sensory quality, therefore high-quality tobacco leaves require maintaining an appropriate balance between carbohydrates and nitrogen compounds (Fang et al., 2025). Organic nitrogen application significantly altered soil microbial community structure, while simultaneously observing increased sugar content, decreased nicotine and total nitrogen content, optimized sugar-to-nicotine ratio, and subsequently improved tobacco quality. These results indicate that microbial community structural changes show close associations with high-quality tobacco production and regulation of key quality components. Based on the clear associations established between the microbiome and crop quality, it is necessary to further investigate the specific microbial compositional changes responsible for these quality improvements.

### 4.3 Microbial community composition changes and the response plateau at 50% organic nitrogen substitution

Organic fertilizer input significantly increased soil organic carbon and nitrogen content as well as carbon source diversity, providing abundant and diverse energy substrates for microorganisms that ranged from simple sugars to complex compounds like cellulose and lignin. By improving the soil physical structure and aeration, the fertilizer fundamentally altered the soil nutritional and physicochemical environment, thereby driving significant shifts in microbial community composition. The establishment of this eutrophic environment promoted the proliferation of copiotrophic microorganisms. Pseudomonas, with its extensive metabolic diversity, was abundantly enriched, indicating rapid response to easily decomposable organic matter and enhanced nutrient cycling efficiency (Preston, 2004; Singh et al., 2022). Fast-growing groups such as Bacteroidota were similarly promoted by increased organic matter input (Schimel and Schaeffer, 2012). Meanwhile, microbial groups with specialized decomposition functions also increased significantly. The enrichment of Actinomycetota was particularly noteworthy. Although this group follows an oligotrophic strategy, it possesses unique advantages in decomposing recalcitrant compounds like cellulose and lignin (Javed et al., 2021), and its increase is crucial for establishing stable long-term carbon pools (Figure 6). Correspondingly, typical oligotrophic indicator groups showed decline. The decrease in Acidobacteriota and Chloroflexota indicated the soil transition from a nutrient-poor to a nutrient-rich state, with significantly enhanced nutrient supply adequacy (Shi et al., 2023; Wongkiew et al., 2022). The reduction in Chloroflexota also reflected that improved soil aeration created a more suitable aerobic environment. Furthermore, changes in nitrogen cycling functional groups reflected shifts in nitrogen supply patterns. The decline of Nitrospirota may be related to abundant organic nitrogen input. Under high-nitrogen conditions, the dominant groups in nitrite oxidation processes may undergo transitions, shifting from Nitrospira to more competitive Nitrobacter (Han et al., 2018), and these changes may affect overall nitrogen use efficiency (Figure 6).

Different organic nitrogen substitution ratios produced differential effects on microbial community composition. The 50% ON and 60% ON treatments showed highly similar microbial compositions, but both differed significantly from the 40% ON treatment (Figure 6). These results indicate that microbial community response reaches a stable state at 50% organic nitrogen substitution, reflecting the nonlinear response characteristics of soil microbial communities to environmental changes (Bardgett and Caruso, 2020; Yu et al., 2025). When the organic nitrogen substitution ratio reached 50%, microbial community structure underwent significant adjustment and gradually tended toward stability, with further increases in organic nitrogen input (60%) having relatively limited effects on community structure. The formation of this response pattern can be attributed to two main factors: First, as organic nitrogen application increased, soil nutrient supply gradually became sufficient, but after reaching a certain level, microbial communities showed saturation effects in nutrient utilization, and community structure no longer underwent significant changes. Second, soil pH changes caused by organic fertilizer application tended to stabilize after reaching a certain degree, and as pH is a key factor affecting microbial community composition (Guo et al., 2023), its stabilization further slowed down continued structural adjustments. This microbiological finding is highly consistent with the aforementioned yield results, explaining from a microbial community structure perspective the biological mechanism behind why continuing to increase organic nitrogen ratios beyond 50% substitution has very limited effects on yield improvement. Despite the overall similarity between the 50% ON and 60% ON treatments, a subtle but ecologically significant difference emerged: the 50% ON treatment retained a higher proportion of Acidobacteriota while having a lower proportion of Pseudomonadota (Figure 6). This reflects a key regulatory advantage. The higher level of Acidobacteriota, an important indicator of carbon pool stability, suggests that the 50% ON treatment maintained relative environmental balance even while promoting active carbon cycling. Concurrently, the moderately lower proportion of the fast-acting Pseudomonadota may have helped prevent overly rapid nutrient release that can lead to a mismatch with crop demand (Anuroopa et al., 2022; Crews and Peoples, 2005). Although numerically small, this fine-tuning of microbial community composition at the 50% produced more significant optimization effects at the functional level.

FAPROTAX functional prediction results showed that the core advantage of the 50% ON treatment lies in the co-enhancement of multiple key functions rather than the extreme strengthening of a single one. Regarding carbon cycling, the 50% ON treatment maintained high abundances for four key functions—hydrocarbon degradation, cellulolysis, aromatic compound degradation, and fermentation—indicating that this treatment could concurrently and efficiently utilize the diverse carbon sources in the organic fertilizer. In contrast, other treatments may have excelled in only some of these functions. For nitrogen cycling, the 50% ON treatment also exhibited a synergistic enhancement of multiple functions, with key processes such as nitrogen fixation, nitrate reduction, and ureolysis all being activated to high levels. This systematic functional optimization across the two core metabolic pathways of carbon and nitrogen cycling provides a solid functional-ecological foundation for the superior performance of the 50% ON treatment in flue-cured tobacco production (Figure 7). It should be noted that while FAPROTAX provides valuable insights into potential microbial functions based on taxonomic composition, it has inherent limitations compared to direct functional analysis methods. Unlike metagenomics, which can directly detect functional genes, or transcriptomics, which reveals actual gene expression levels, FAPROTAX relies on predictive modeling with main limitations being that this method assumes all members within the same taxonomic group possess identical functions (although functions may not be completely conserved within taxonomic groups), and prediction effectiveness is heavily dependent on the depth and accuracy of taxonomic identification, with functional annotation primarily based on accurate identification at the genus and order levels (Sansupa et al., 2021). Despite these inherent limitations of predictive methods, FAPROTAX still provides an effective analytical framework for understanding microbial functional potential and identifying functional differences between treatments, particularly offering important value in revealing functional trends and relative change patterns.

### 4.4 Effects of organic nitrogen substitution on microbial network characteristics

Microbial network analysis further elucidated the regulatory mechanisms of organic nitrogen on the soil microbial ecosystem from the perspectives of stability and functional coordination. This study found that organic nitrogen treatments significantly enhanced the complexity of the soil microbial network, as evidenced by an increase in the number of edges and network density (Tables 2, 3). In ecology, a more complex network generally implies greater community stability, higher resistance to disturbances, and more efficient overall function. The increased number of connections and tighter relationships among nodes suggest that more complex collaborative patterns may have formed among the microorganisms. Dense local connections within a network often reflect cross-feeding, diverse metabolic pathways, or similar niche preferences, indicating efficient resource utilization and functional collaboration within the community (Poisot and Gravel, 2014). These complex collaborative patterns help enhance ecosystem adaptability through redundant functional pathways and promote efficient nutrient cycling (Shade et al., 2012; Matchado et al., 2021). In addition to increased complexity, a significant decrease in the modularity index was another key finding (Tables 2, 3). A lower modularity index indicates that more cross-module connections were established between previously independent functional modules, forming a more integrated functional network (Maurice et al., 2024). Notably, organic nitrogen treatments generally weakened positive correlations and strengthened negative correlations within the soil microbial network, a phenomenon most pronounced in the 50% ON treatment (Tables 2, 3; Supplementary Figures S4, S5). The 50% ON treatment simultaneously exhibited the highest network complexity, the most negative correlations, and the lowest modularity index. These unique network structural characteristics may promote fine-scale functional differentiation within the community, where different taxa achieve optimized niche partitioning through interactions, potentially improving overall resource utilization efficiency (Coyte et al., 2015; Yu et al., 2024). This network structural pattern shows consistent trends with the superior performance of this treatment in terms of yield and quality, providing an additional microbial ecological perspective for the advantages of the 50% ON treatment. However, the specific relationship between network structure and functional performance still requires further research and verification.

## 5 Conclusion

This study systematically evaluated the effects of different fertilization treatments on tobacco agronomic traits, yield, chemical composition, and sensory quality, confirming that 50% organic nitrogen substitution ratio was the optimal fertilization scheme. This treatment achieved yield increases of 63.4 and 67.8% at two experimental sites respectively, and showed optimal performance in tobacco leaf chemical quality and sensory quality. Soil microbial community analysis further revealed the biological basis of organic nitrogen action. Organic nitrogen provided important ecological support for tobacco yield and quality improvement by enhancing microbial community richness and diversity, restructuring community composition, optimizing carbon and nitrogen cycling functions, and increasing network complexity. Among these, microbial community response reached a stable state at 50% organic nitrogen substitution, showing optimal performance in community structure and functional coordination. This study revealed the effects of different organic nitrogen substitution ratios on tobacco yield and quality and the response characteristics of soil microbial communities, preliminarily elucidated the microbial ecological mechanisms of 50% organic nitrogen substitution, and provided theoretical reference for precision tobacco fertilization based on microbial regulation.

## Data Availability

The datasets presented in this study can be found in online repositories. The names of the repository/repositories and accession number(s) can be found at: https://www.ncbi.nlm.nih.gov/, PRJNA858879.

## Footnotes

^1^ https://cloud.majorbio.com
