# Supplementary material for: Microbial response mechanisms of organic nitrogen substitution for optimizing tobacco yield and quality: the key role of 50% organic nitrogen
Source: Front Microbiol. 2025 Oct 9;16:1698745. doi: 10.3389/fmicb.2025.1698745 (PMC12546161; doi:10.3389/fmicb.2025.1698745)
Supplement: Supplementary file 1 [file Data_Sheet_1.docx]

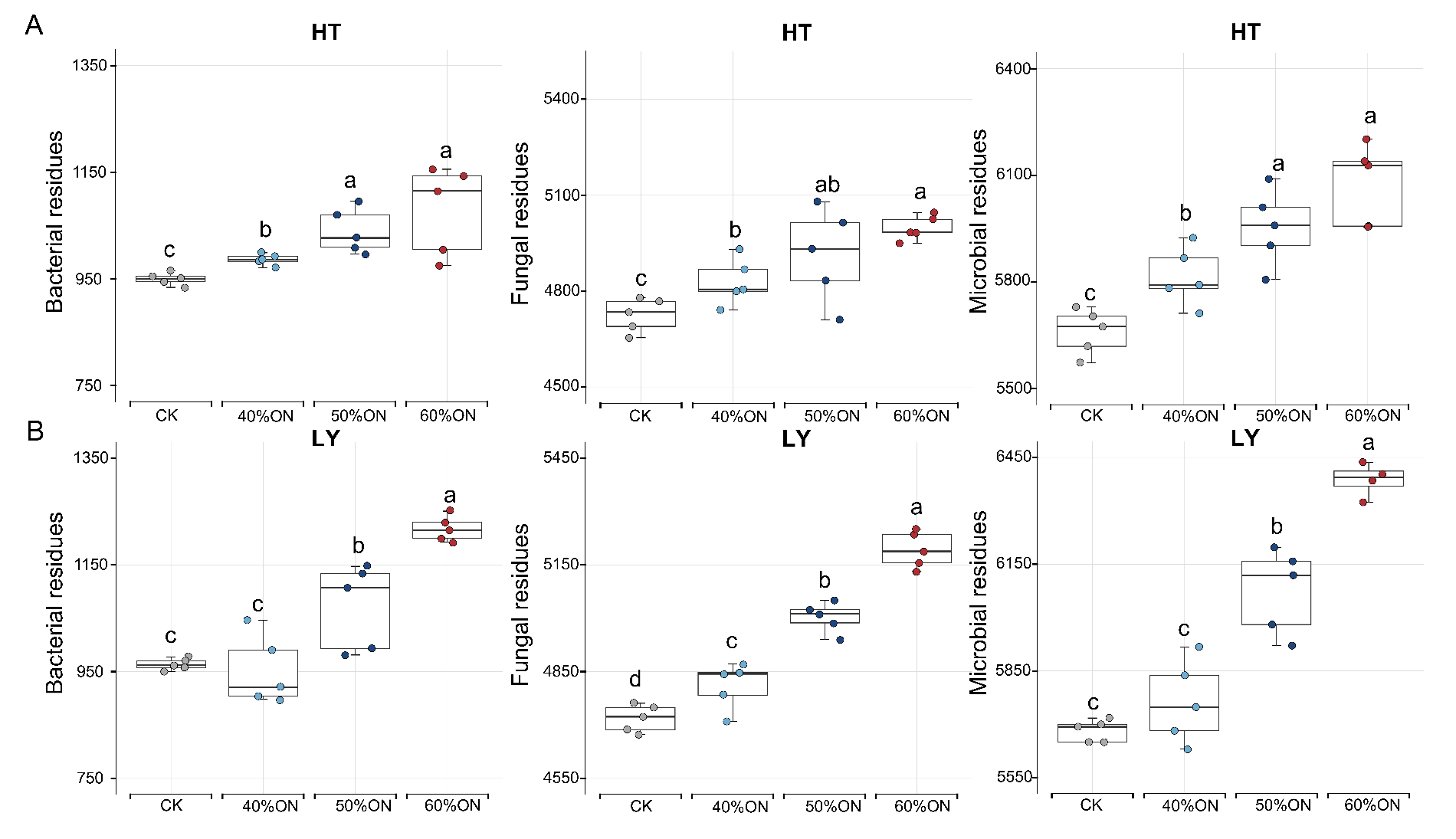


Figure S1. Effects of different nitrogen treatments on bacterial, fungal, and total microbial carbon residue content in soil. (A) HT experimental site, (B) LY experimental site.


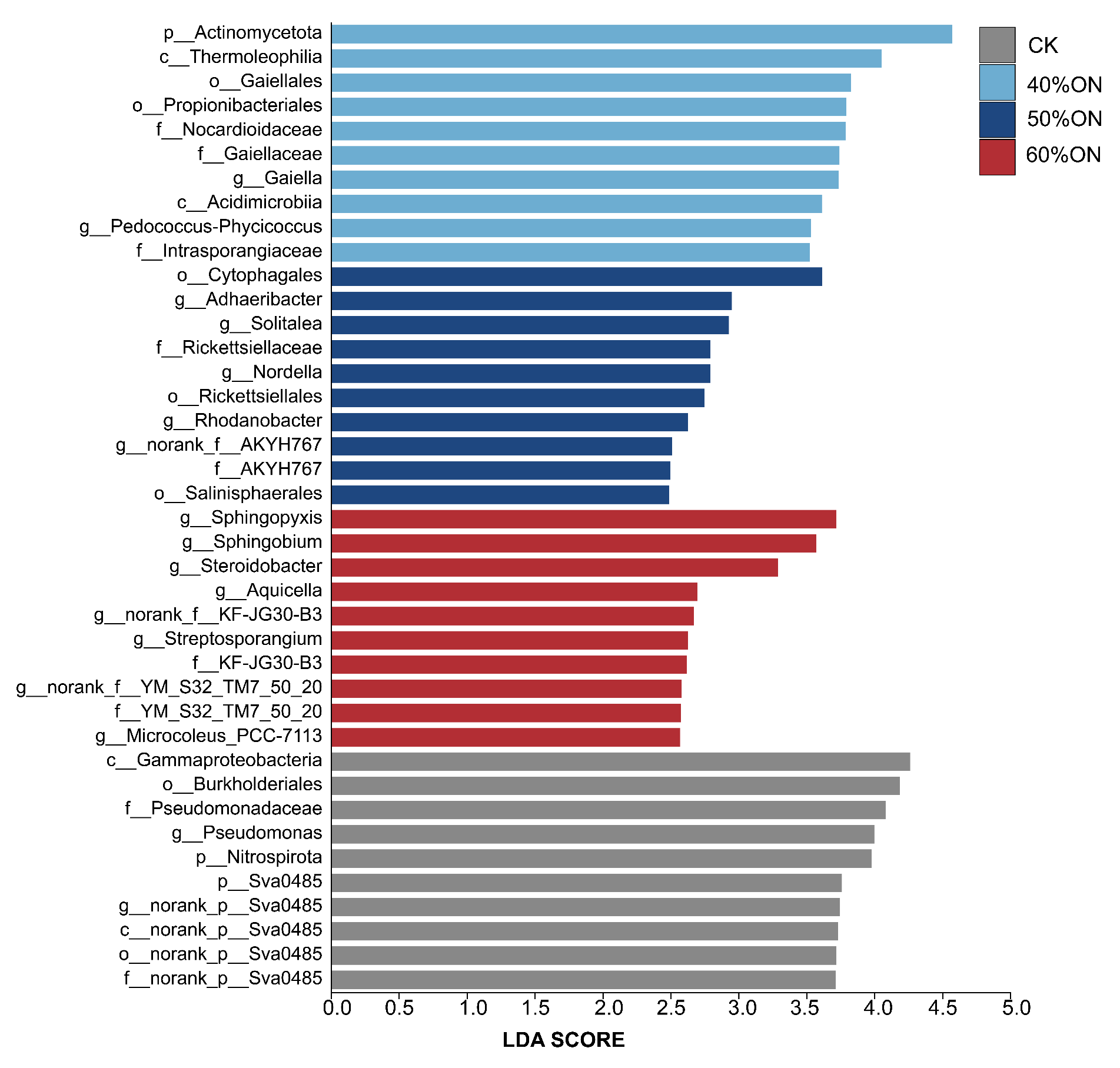


Figure S2. Effects of different organic nitrogen treatments on microbial biomarkers in soil at the HT experimental site. Different colored bars represent species with higher relative abundance in different treatment groups (LDA score ≥ 3.0).


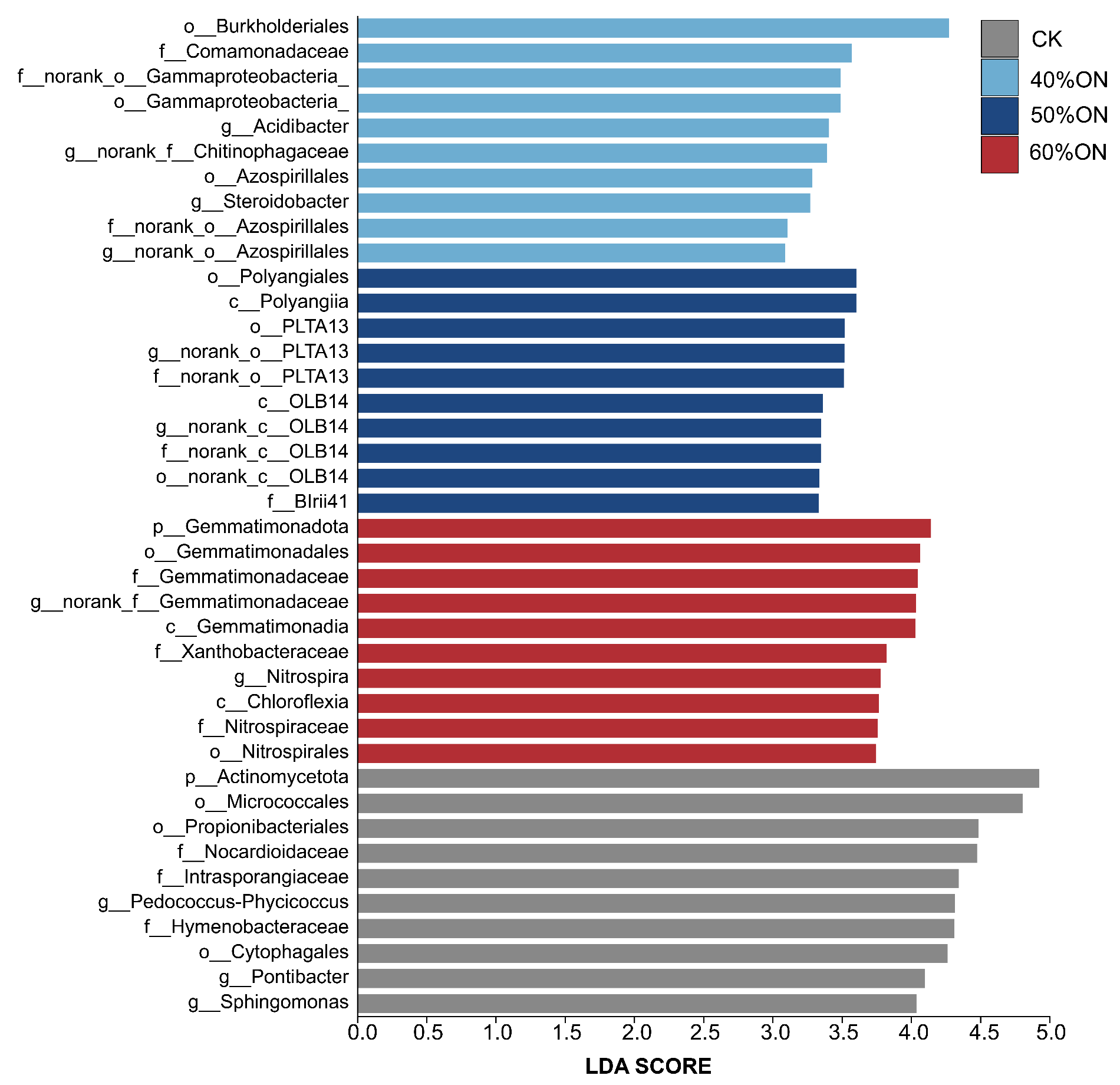


Figure S2. Effects of different organic nitrogen treatments on microbial biomarkers in soil at the LY experimental site. Different colored bars represent species with higher relative abundance in different treatment groups (LDA score ≥ 3.0).


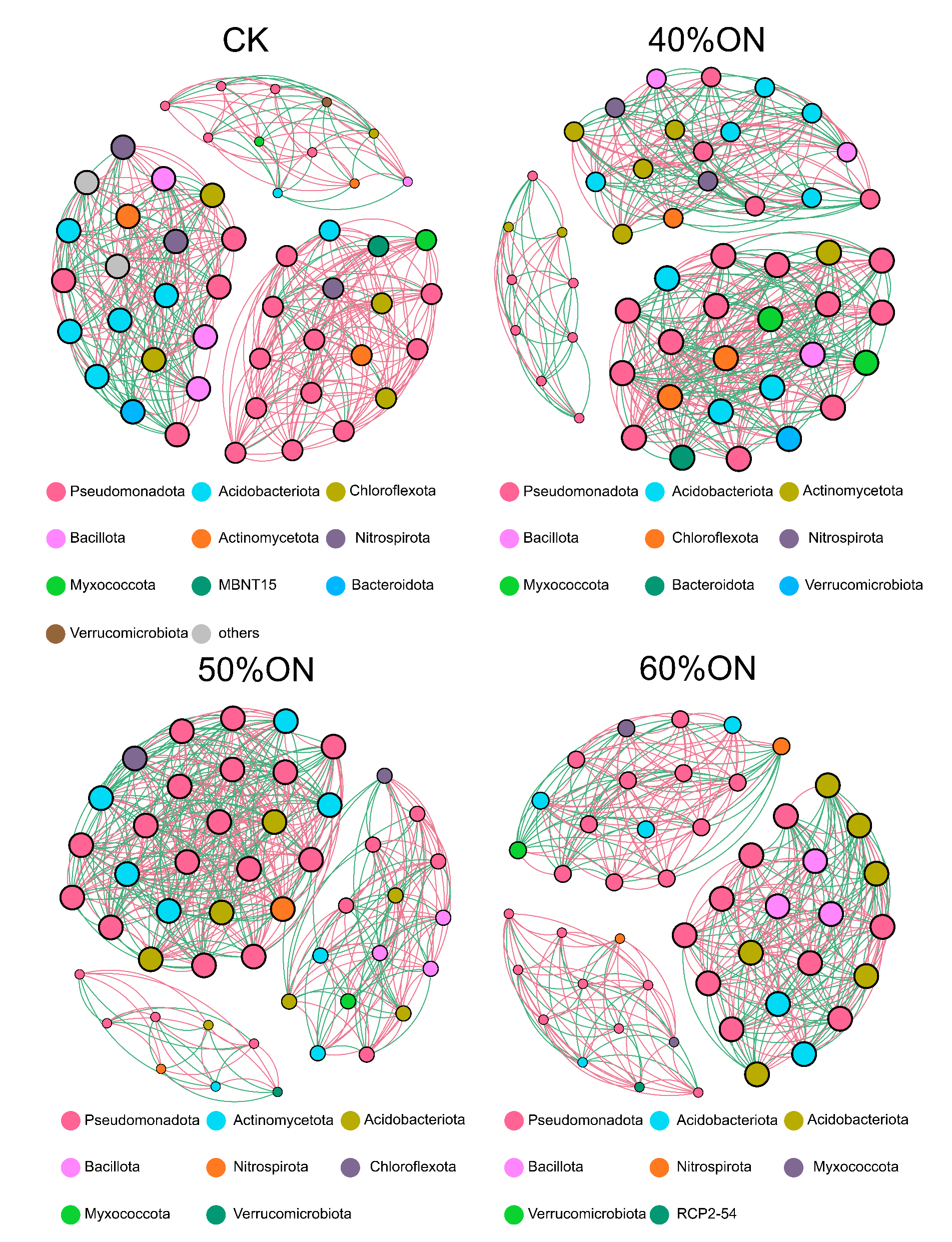


Figure S4. Effects of different organic nitrogen treatments on soil bacterial co-occurrence networks at the HT experimental site. Different colored nodes represent species at different phylum levels.


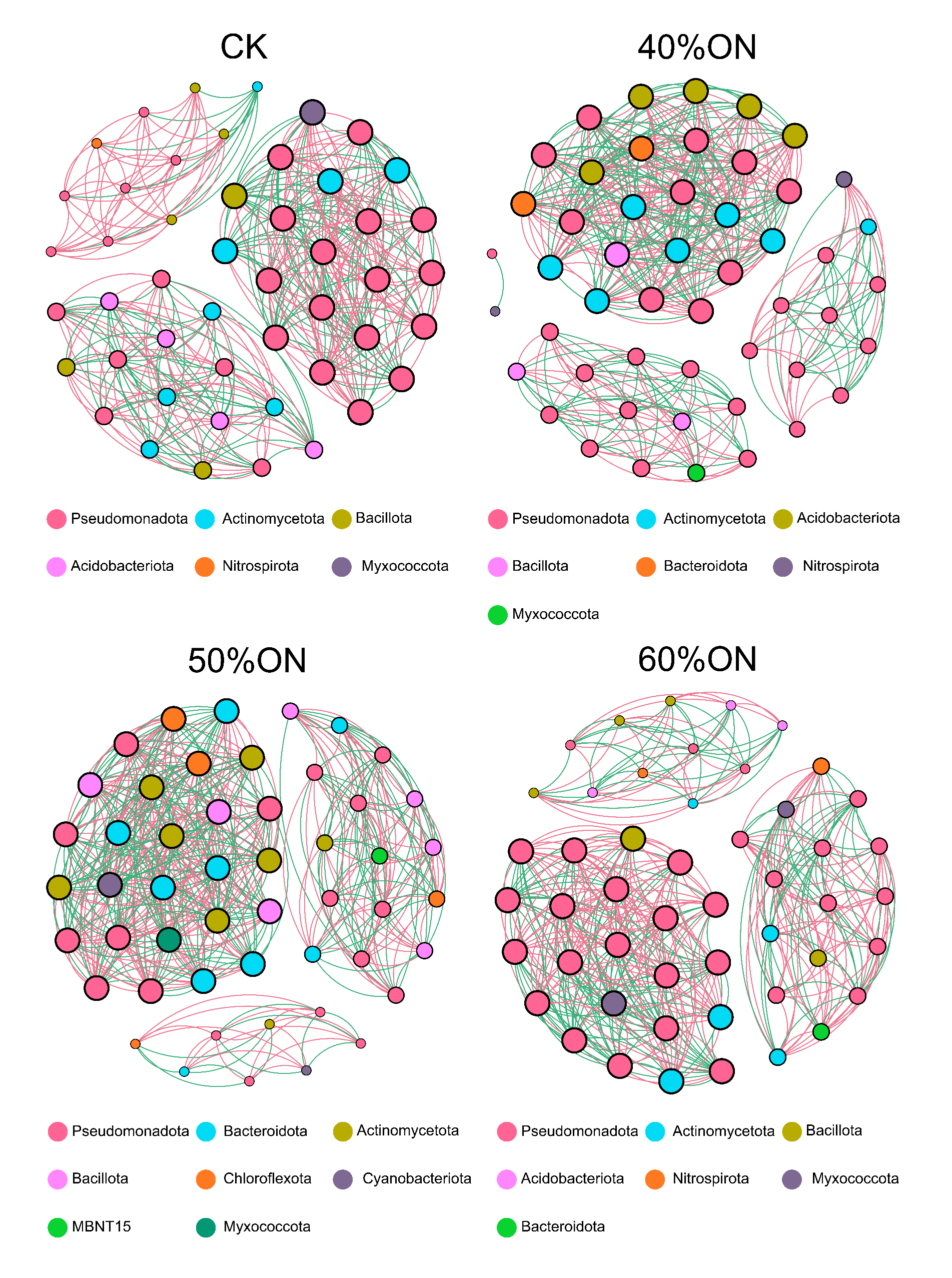


Figure S4. Effects of different organic nitrogen treatments on soil bacterial co-occurrence networks at the LY experimental site. Different colored nodes represent species at different phylum levels.
